# Exploring profiles of fathers integrating food and physical activity parenting practices

**DOI:** 10.1017/S1368980025000278

**Published:** 2025-03-17

**Authors:** John A Jimenez-Garcia, Louise C Mâsse, Robert L Newton, Salma M Musaad, Alicia Beltran, Teresia M O’Connor

**Affiliations:** 1 USDA/ARS Children’s Nutrition Research Center, Department of Pediatrics, Baylor College of Medicine, Houston, TX, US; 2 BC Children’s Hospital Research Institute, School of Population and Public Health, University of British Columbia, Vancouver, BC, Canada; 3 Population and Public Health, Pennington Biomedical Research Center, Baton Rouge, Louisiana, USA

**Keywords:** Father–child, Physical activity, Nutrition, Parenting practices, Latent profile analysis, Social determinants of health, Co-parenting

## Abstract

**Objective:**

This study aims to identify fathers’ profiles integrating food parenting practices (FPP) and physical activity parenting practices (PAPP).

**Design:**

We analysed cross-sectional data. The fathers completed the reduced FPP and PAPP item banks and socio-demographic and family dynamics (co-parenting and household responsibility) questionnaires. We identified fathers’ profiles via latent profile analysis. We explored the influence of social determinants, child characteristics and family dynamics on fathers’ profiles using multinomial logistic regression.

**Setting:**

Online survey in the USA.

**Participants:**

Fathers of 5–11-year-old children.

**Results:**

We analysed data from 606 fathers (age = 38 ± 8·0; Hispanic = 37·5 %). Most fathers self-identified as White (57·9 %) or Black/African American (17·7 %), overweight (41·1 %) or obese (34·8 %); attended college (70 %); earned > $47 000 (62·7 %); worked 40 hrs/week (63·4 %) and were biological fathers (90·1 %). Most children (boys = 55·5 %) were 5–8 years old (65·2 %). We identified five fathers’ profiles combining FPP and PAPP: (1) *Engaged Supporter Father* (*n* 94 (15·5 %)); (2) *Leveled Father* (*n* 160 (26·4 %)); (3) *Autonomy-Focused Father* (*n* 117 (19·3 %)); (4) *Uninvolved Father* (*n* 113 (18·6 %)) and (5) *Control-Focused Father* (*n* 122 (20·1 %)). We observed significant associations with race, ethnicity, child characteristics, co-parenting and household responsibility but not with education level, annual income or employment status. We observed significant pairwise differences between profiles in co-parenting and household responsibility, with the *Engaged Supporter Father* presenting higher scores in both measures.

**Conclusions:**

Understanding how fathers’ FPP and PAPP interact can enhance assessments for a comprehensive understanding of fathers’ influences on children’s health. Recognising the characteristics and differences among fathers’ profiles may enable tailored interventions, potentially improving children’s health trajectories.

Insufficient physical activity is a leading risk factor for non-communicable disease mortality, contributing to ∼5·3 million deaths worldwide^(1,2)^. Unhealthy eating (e.g. poor eating behaviour and high intakes of energy-dense, nutrition-poor foods) represents another significant risk factor for non-communicable diseases and related conditions^(2,3)^. The combined impact of physical inactivity and unhealthy eating can accelerate the onset and coexistence of various comorbidities, including cardiovascular (e.g. hypertension), metabolic (e.g. type 2 diabetes) and mental health (e.g. depression) conditions^(1,2)^.

From an ecological perspective, children’s physical activity, eating behaviour and dietary intake are influenced by individual factors such as age and sex, as well as by social and environmental factors, like culture, family, school and public health^(4)^. The family environment plays a crucial role in children’s health trajectories, with parents acting as key influencers^(5)^. Parents play a central role in preventing the development of unhealthy behaviours (e.g. increased sedentary time) and health-enhancing behaviours (e.g. physical activity) during childhood^(6)^. Within the family micro-environment, parents use various parenting practices to influence and interact with their children. These parenting practices are goal-directed, context-specific, child-rearing strategies parents use to influence their children’s behaviours^(7)^. For instance, parental encouragement, logistic support and co-participation have been associated with children’s physical activity levels^(8)^. Similarly, parental monitoring and modelling, food accessibility and child involvement have been associated with children’s dietary intake^(9,10)^. Parenting practices are thought to be easier to target and change in interventions^(11)^, but they can be influenced by various factors. Co-parenting^(12)^, household responsibility^(13)^ and child characteristics impact family dynamics, which in turn affects parents’ behaviours. Furthermore, social determinants of health (i.e. socio-economic status, race and ethnicity) shape parenting practices, family dynamics and children’s health environment^(14)^.

Two key components of parenting practices for children’s health and well-being are food parenting practices (FPP) and physical activity parenting practices (PAPP)^(10,15)^. FPP represent a wide array of techniques and behaviours used by parents to influence children’s eating behaviour and dietary intake^(10)^. Similarly, PAPP are the techniques or behaviours used to influence children’s physical activity^(15)^. FPP and PAPP have systematic conceptual frameworks and have been operationalised in the form of item banks^(16–18)^. In previous work based on expert input, FPP and PAPP mapped across sub-factors in three parenting domains: autonomy promotion, structure and control^(10,15)^. Although FPP and PAPP have been widely studied individually, their combined use has not been concurrently explored, despite many interventions and public health programs targeting both physical activity and nutrition^(19,20)^. Understanding how FPP and PAPP work together can enhance the development and effectiveness of interventions and programmes aimed at promoting healthy lifestyles among children.

Although similarities between fathers’ and mothers’ parenting practices exist, evidence suggests that each parent’s practices uniquely influence and are differently associated with children’s health behaviours^(11)^. The role and influence of fathers in shaping children’s health through both physical and eating behaviours are still understudied, particularly among under-represented populations (e.g. Hispanics, Black/African American)^(21–23)^. Previous studies have identified parents’ profiles using either FPP or PAPP^(8,24)^; but none have exclusively examined fathers’ profiles based on both FPP and PAPP, highlighting two gaps: the lack of focus on fathers and the absence of integrated analysis combining both FPP and PAPP.

Understanding how fathers’ FPP and PAPP interact and influence each other can provide a more comprehensive picture of the factors shaping children’s health behaviours. By considering factors like social determinants of health, family dynamics and child characteristics, and examining how they intersect with fathers’ FPP and PAPP, we can help address existing knowledge gaps, particularly in under-represented communities. This study’s objectives are threefold: (1) identify fathers’ profiles integrating FPP and PAPP using latent profile analysis (LPA), (2) explore the influence of social determinants of health, child characteristics and family dynamics (i.e. co-parenting and household responsibility) in the profiles and (3) examine profile differences in fathers’ co-parenting and household responsibility. Our overarching goal is to enhance the understanding of how fathers’ parenting practices influence children’s health environments, ultimately contributing to public health nutrition and physical activity by informing targeted efforts for improving children’s health outcomes and fostering healthier family environments.

## Methods

### Study design

This study fulfilled the secondary aim of a cross-sectional online study, which assessed the psychometric properties of the reduced versions of the FPP and PAPP item banks in a diverse sample of fathers (Musaad et al. manuscript under review). The participants completed the online questionnaires between April 2018 and July 2019. We used the STROBE guidelines to report this secondary analysis on cross-sectional data^(25)^.

### Participants

We recruited participants in the United States using printed and electronic notices, posters and flyers distributed in multiple settings including community centres, clinics, hospitals, research centres, fatherhood organisations, local businesses, worksites, child-care centres, schools, social media and newsletters. The participants met the following inclusion criteria: (1) being a father of a child aged 5–11 years, (2) the child lived with the father at least 50 % of the time and (3) the child was healthy and could participate in regular physical activity (e.g. physical education, organised sport) and could eat a regular diet. We reached 1949 contacts who were interested in participating in the study and directed them to the online survey that could be completed in either English or Spanish. Of those interested contacts, 1035 consented to participate in the study. An online screener verified that the participants met the inclusion criteria, and 890 passed the screening process. Reasons for excluding participation included as follows: (1) thirty-three participants were not fathers of a 5–11-year-old child, (2) sixteen children did not live at least 50 % of the time with the father, (3) seven children were either not able to participate in regular physical activity or eat regular foods and (4) eighty-nine were removed after data cleaning (twenty-two duplicates, sixty-five female respondents, two non-compliant participants). Out of the 890 participants, 284 had partial data and 606 completed the questionnaires. We nominally compensated ($15) the fathers’ participation.

### Measures

The online survey comprised 100 questions: eighteen on demographics, thirty on FPP, thirty-six on PAPP and sixteen on family dynamics.

#### Demographics

We collected self-reported demographic data including age, height, weight, marital status, child age, child biological sex at birth, relationship with the child, race and ethnicity. The categories for race were Asian, Black/African American, Mixed/Multiple races, Native American or Alaskan Native, Native Hawaiian or Pacific Islander, Other and White. The categories for ethnicity were Hispanic, Latino, Mexican American, or non-Hispanic, non-Latino and non-Mexican American. We measured socio-economic status using education level, employment situation and annual household income, which was based on the following cut-offs: < $25 000, $25 000–$46 000, > $47 000. We determined the lower cut-off based on the poverty threshold for a family of four^(26,27)^, considering the average Hispanic household size of 3·25. Moreover, we determined the upper cut-off based on the median income of Hispanic households in 2016^(28)^ and the threshold for the middle class in the USA in 2016^(29)^. We also obtained information regarding the partner’s employment status.

#### Parenting practices

We used the reduced versions of the FPP and PAPP item banks^(17,18)^, validated through confirmatory factor analysis (CFA) (Musaad et al. manuscript under review). Although the full version of the FPP and PAPP item banks had previous validity evidence, we tested the psychometric properties of the reduced versions to minimise participant burden and simplify administration. The FPP and PAPP item banks encompass three parenting domains: autonomy promotion, structure and control^(10,15)^. Autonomy promotion refers to the ways parents, through support, promote developmentally appropriate choices and decisions of children’s own behaviours. Structure relates to the parents’ attempts to promote child proficiency in a non-directive way by organising the child’s environment. Control refers to the parents’ attempts to impose their will or direct children’s behaviours without considering children’s desires. In the first aim of this study (Musaad et al. manuscript under review), we focussed on one or two most relevant constructs (i.e. latent factors) within each domain to streamline the item banks for future clinical trials or father-targeted programs. Regarding FPP, one construct (i.e. child involvement) for the autonomy promotion domain, two constructs (i.e. covert control and modelling) for the structure domain and one construct (i.e. threats and bribes) for the control domain were included in the CFA. Regarding PAPP, two constructs (i.e. parental involvement and praise) for the autonomy promotion domain, two constructs (i.e. co-participation and modelling) for the structure domain, and two constructs (i.e. guilt and pressure) for the control domain were included in the CFA. All items in the constructs asked how often the father performed the practice in the past month using a five-point scale. The factor structures of both the FPP and PAPP were supported by the CFA and invariance testing for Hispanic and non-Hispanic fathers, as well as the instrument being completed in English or Spanish. Factor loadings, correlation coefficients (FPP range 0·59–0·79 and PAPP range 0·74–0·87) and fit indices were consistent with the values reported in the first aim of this study (Musaad et al. manuscript under review).

#### Co-parenting alliance and division of household labour (PEW)

We used the co-parenting alliance questionnaire which consists of nine items scored using a 5-point scale (Not at all (1) – Not very often (2) – Sometimes (3) – Fairly often (4) – Almost always (5)). This questionnaire assesses the parenting aspects of a couple’s relationship during the childrearing process^(12)^. We used this instrument to capture father’s perception of how cooperative, communicative and respectful he considered he and his partner were regarding caring for their child^(12)^. Furthermore, we used the PEW questionnaire to assess fathers’ perceptions regarding the division of labour in households^(13)^. The questionnaire comprises five items and uses a nominal scale. For pragmatism in our analysis, we assigned weights to the answer options as follows: ‘I do more’ = 2 points, ‘Share about equally’ = 1 point, ‘Spouse/partner/child’s other parent/children’s other parent does more’ = 0. Answer options ‘Other’ and ‘Don’t know/Refused’ (*n* 47, 7·8 % of the sample (*n* 606)) were excluded from the analysis as they did not provide information about the partner. We totalled all scores to calculate an index (ranging from 0 to 10) based on the five items.

### Statistical analysis

#### General considerations

We conducted our statistical analysis in four stages using R (version 4.3.1; R-core team, 2021): (1) compute demographic statistics; (2) compute factor scores; (3) derive latent profiles (model specification, fit, and evaluation) and (4) analyse the profiles (e.g. profile membership and differences).

#### Computing factor scores through confirmatory factor analysis

We used factor scores as the input for the LPA model. We used the lavaan library and the factor structure tested in the first aim of this study (Musaad et al. manuscript under review) to compute factor scores from the reduced versions of the FPP and PAPP item banks with our slightly reduced sample. We used Bartlett’s approach to compute the factor scores^(30)^. Bartlett’s approach is a refined method that produces unbiased estimates that most likely represent the ‘true’ factor scores by using maximum likelihood. Bartlett’s approach considers the factor structure and both what is shared between the observable item and the factor (i.e. shared variance) and what is not measured (e.g. uniqueness)^(30)^. We obtained standardised scores (ranging from +3 to –3) with mean zero and variance reflecting the squared multiple correlation between items and factor^(30)^. The computed factor scores were significantly correlated with their corresponding raw scores (*r* = (0·88–0·99), *P* < 0·001).

#### Latent profile analysis

##### Model specification

We conducted our LPA using the tidyLPA library, which is built on mclust. We used the Expectation–Maximisation algorithm to iteratively maximise the likelihood function to estimate parameters^(31)^. We used the class varying diagonal parametrisation (model 2 in tidyLPA) where we allowed the variances to be freely estimated across profiles but the covariances were constrained to zero^(31)^. We used the tidyLPA model 2 because we provided factor scores from a theoretically driven and empirically tested factor structure derived from a CFA (Musaad et al. manuscript under review). We expected the factors to vary, while the covariances are expected to be low due to confirmatory factor structure. We looked for a combination of model fit and parsimony, and the model 2 provided enough flexibility to identify profiles while maintaining a balance between complexity and simplicity considering the expected differences between FPP and PAPP^(32)^.

##### Model evaluation

We selected the ‘best’ fitting profile solution based on statistical fit indices and theoretical and content-related considerations^(32)^. To assess how well a model fit the data while balancing complexity, we used information criteria such as the Bayesian Information Criterion and Akaike Information Criterion, choosing the model with the lowest values. We evaluated model performance by measuring classification confidence levels using entropy, where higher values indicate greater certainty. To test whether adding an extra profile improved the model, we applied the bootstrapped likelihood ratio test. Additionally, we qualitatively assessed whether the latent profiles provided meaningful insights and aligned with theoretical expectations. We examined the content of each profile to ensure it contributed to a deeper understanding of the constructs and assigned appropriate labels.

#### Multinomial logistic regression

After identifying the optimal profile solution, we conducted further statistical tests to contextualise the LPA results. We applied multinomial logistic regression to explore the associations between the fathers’ profiles with the social determinants of health (i.e. annual income, employment status, education level, race and ethnicity), child characteristics (i.e. child age and child sex) and family dynamics (i.e. co-parenting and household responsibility). In the variable race, we collapsed the categories of Native American or Alaskan Native, Native Hawaiian or Pacific Islander and Multiple/Mixed into the Other/Mixed categories due to small sample sizes as it may affect convergence of the regression model. Similarly, we dichotomised the variable education level in university degree (or higher) and non-university degree. We determined the reference categories in two different ways: (1) we used the category with the higher proportion for annual income, employment status and education level and (2) following recommended standards in reporting to enhance fairness, equity, consistency and clarity, we listed the categories in alphabetical order for race, ethnicity, child age and child sex^(33)^. We sequentially included each group of variables in a stepwise manner and assessed the significance of the likelihood ratio test to determine its inclusion in the model. To interpret the results, we computed OR by exponentiating the log odds obtained from the logistic regression. Furthermore, we tested for differences between profiles for co-parenting alliance scores and household responsibility index scores. We used the Kruskal–Wallis test for an overall comparison and performed pairwise Wilcoxon tests with Bonferroni correction to assess specific group differences. We conducted all statistical analyses with a significance level of 0·05.

## Results

### Demographics

We analysed data from 606 fathers (age = 38·05 ± 8·06; Hispanic = 37·46 %). Most fathers self-identified as White (57·92 %) or Black/African American (17·66 %); were overweight (41·1 %) or obese (34·8 %); attended college (70 %); earned > $47 000 (62·71 %); worked 40 h/week (63·37 %) and were biological fathers (90·10 %). Most children (boys = 55·28 %) were 5–8 years old (65·18 %). See participants’ demographics in Table 1.

**Table 1.** Proportions of demographic characteristics of the sample

Variable	Engaged supporter father (*n* 94)		Leveled father (*n* 160)		Autonomy-focused father (*n* 117)		Uninvolved father (*n* 113)		Control-focused father (*n* 122)		All (*n* 606)	
%	15·51		26·4		19·31		18·65		20·13		100	
	Mean	sd	Mean	sd	Mean	sd	Mean	sd	Mean	sd	Mean	sd
Father age (years) (mean (sd))	36·06	8·63	38·14	7·25	40·15	7·85	37·43	7·88	38·02	8·63	38·05	8·06
Father height (m) (mean (sd))	1·74	0·27	1·77	0·16	1·77	0·08	1·77	0·08	1·73	0·24	1·76	0·18
Father weight (kg) (mean (sd))	94·41	22·29	89·64	20·31	90·56	18·38	91·89	17·34	88·59	19·46	90·76	19·60
Father BMI (kg/m^2^) (mean (sd))	30·11	7·44	28·29	5·78	28·86	4·84	29·25	4·97	28·70	6·78	28·94	5·98
	%		%		%		%		%		%	
Marital status (%)												
Married/common	79·79		87·5		91·45		89·38		84·43		86·8	
Other	20·21		12·5		8·55		10·62		15·57		13·2	
Relationship to child (%)												
Biological father	88·3		92·5		88·89		92·04		87·7		90·1	
Stepfather	10·64		5		7·69		3·54		8·2		6·77	
Adoptive or foster father	1·06		0·63		3·42		4·42		2·46		2·31	
Other father figure	0		1·88		0		0		1·64		0·83	
Child age (%)												
5–8 years	68·09		65·63		67·52		75·22		50·82		65·18	
9–11 years	31·91		34·38		32·48		24·78		49·18		34·82	
Child sex (%)												
Boy	70·21		56·25		52·14		48·67		51·64		55·28	
Girl	29·79		43·75		47·86		51·33		48·36		44·72	
Income (%)												
Less than $25 000	17·02		10·63		9·4		13·27		20·49		13·86	
Between $25 000 and $47 000	24·47		21·88		17·95		23·01		30·33		23·43	
More than $47 000	58·51		67·5		72·65		63·72		49·18		62·71	
Education level (%)												
Some school	9·57		6·25		7·69		12·39		15·57		10·07	
High school or technical school	26·6		18·75		9·4		26·55		20·49		19·97	
Some college	21·28		20		23·08		14·16		18·03		19·31	
College graduate	23·4		26·88		32·48		20·35		29·51		26·73	
Postgraduate	19·15		28·13		27·35		26·55		16·39		23·93	
Father employment status (%)												
Not currently employed	8·51		6·88		9·4		9·73		13·93		9·57	
Part time (< 40 h/week)	13·83		6·88		5·13		7·96		9·02		8·25	
Full time (40 h/week)	58·51		60		71·79		60·18		66·39		63·37	
More than full time (> 40 h/week)	19·15		26·25		13·68		22·12		10·66		18·81	
Partner employment status (%)												
Not currently employed	22·34		31·88		27·35		29·2		22·95		27·23	
Part time (< 40 h/week)	17·02		10		13·68		19·47		18·03		15·18	
Full time (40 h/week)	43·62		40		52·14		37·17		38·52		42·08	
More than full time (> 40 h/week)	10·64		10		5·13		6·19		6·56		7·76	
Not available	6·38		8·13		1·71		7·96		13·93		7·76	
Race (%)												
Black/African American	24·47		15·63		12·82		14·16		22·95		17·66	
Asian	5·32		7·5		5·13		4·42		11·48		6·93	
Other/Mixed	20·21		15		17·95		16·81		18·85		17·49	
White	50		61·88		64·1		64·6		46·72		57·92	
Ethnicity (%)												
Hispanic	44·68		32·5		25·64		41·59		45·9		37·46	
Non-Hispanic	55·32		67·5		74·36		58·41		54·1		62·54	

sd: Standard Deviation.

### Estimation and description of latent profiles

**Figure 1.**
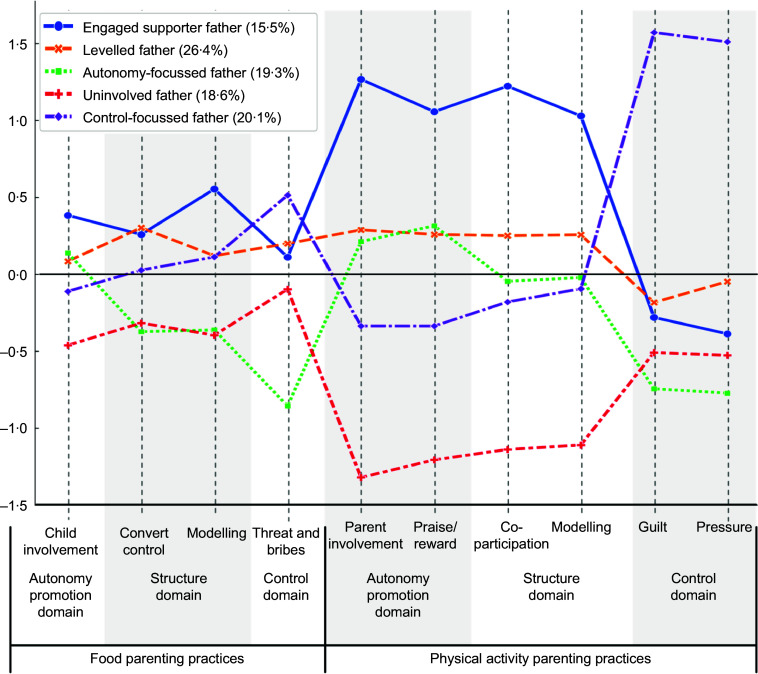
Fathers’ profiles ordered by parenting practices.

The fit indices did not suggest a single and definitive solution for the LPA as reported in other studies using a similar approach^(8)^. After reviewing the fit indices, visually inspecting the models for four to eight profiles and discussing whether new profiles enhanced our understanding of the constructs, we selected the five-profile model (Figure 1) based on a high representation of fathers (> 15 %) in each profile and its theoretical relevance. Based on the literature and the observed differences, we labelled the profiles as follows: *Profile 1, the ‘Engaged Supporter Father*’ (*n* 94, 15·5 %) presents a supportive approach in both FPP and PAPP. In FPP, the father encourages the child’s independence with guidance and role modelling. Bribes and threats are used relatively sparingly. In PAPP, the father actively involves the child and sets a positive example. The use of guilt or pressure is low. This father presents the highest values in autonomy promotion and structure compared with the other profiles. *Profile 2, the ‘Leveled Father’* (*n* 160, 26·4 %), employs a balanced and supportive approach in FPP and PAPP, with similar scores across all parenting practices. This father does not outperform or underperform any other profile in any factor or domain. *Profile 3, the ‘Autonomy-Focused Father’* (*n* 117, 19·3 %). This father uses mostly autonomy promotion for FPP and PAPP, while control in FPP and PAPP is the lowest among all the profiles. *Profile 4, the ‘Uninvolved Father’* (*n* 113, 18·6 %), exhibits the most limited involvement and structure among profiles in FPP and PAPP, with low control. *Profile 5, the ‘Control-Focused Father’* (*n* 122, 20·1 %), the father has the highest use of threats and bribes for FPP and PAPP, with relatively low autonomy promotion and structure compared with the other profiles. Of note, the variability in standardised scores is greater for the PAPP than for the FPP for the five profiles of fathers in this sample. See the results for parenting practices factors in Table 2.

**Table 2.** Mean and standard deviations of FPP and PAPP factors in fathers’ profiles

Variable	Engaged supporter father (*n* 94)		Leveled father (*n* 160)		Autonomy-focused father (*n* 117)		Uninvolved father (*n* 113)		Control-focused father (*n* 122)		All (*n* 606)	
Food parenting practices	Mean	sd	Mean	sd	Mean	sd	Mean	sd	Mean	sd	Mean	sd
Autonomy promotion domain												
Child involvement factor	3·04	0·64	2·85	0·61	2·84	0·45	2·54	0·66	2·83	0·68	2·82	0·68
Structure domain												
Covert control sub-factor	3·14	0·9	3·23	0·79	2·79	0·97	2·8	0·91	3·04	0·94	3·01	0·91
Modeling sub-factor	3·59	0·77	3·25	0·71	3·01	0·92	2·95	0·91	3·35	0·73	3·22	0·83
Control domain												
Treats and bribes factor	2·14	0·72	2·18	0·64	1·64	0·63	2·05	0·7	2·35	0·69	2·08	0·71
Physical activity parenting practices												
Autonomy promotion domain												
Child involvement sub-factor	4·54	0·52	3·83	0·56	3·87	0·57	2·85	0·81	3·41	0·83	3·68	0·85
Praise sub-factor	4·86	0·49	4·26	0·57	4·38	0·59	3·24	1·13	3·78	0·93	4·09	0·93
Structure domain												
Co-participation sub-factor	4·16	0·67	3·47	0·66	3·35	0·58	2·71	0·78	3·28	0·78	3·37	0·82
Modelling sub-factor	4·13	0·67	3·68	0·6	3·54	0·58	2·94	0·81	3·55	0·74	3·56	0·76
Control domain												
Guilt sub-factor	1·55	0·6	1·62	0·59	1·17	0·42	1·36	0·54	2·57	1·03	1·67	0·83
Pressure sub-factor	1·61	0·57	1·89	0·55	1·4	0·45	1·58	0·55	2·62	0·75	1·84	0·72

sd: Standard Deviation; Scores ranged from 0 to 5.

### Association between profiles, social determinants of health, child characteristics and family dynamics

We included all intended blocks of variables (i.e. social determinants of health; e.g. annual income, employment status, education level, race and ethnicity), child characteristics (i.e. child age and child sex) and family dynamics (i.e. co-parenting and household responsibility)) in the multinomial logistic regression model. After adding each block of variables, each likelihood ratio test was statistically significant (*P* < 0·001), suggesting an adequate level of improvement and significance.

We observed significant associations in the multinomial regression analysis. When all other variables are held at their reference values, fathers are more likely to belong to the *Leveled Father*, *Uninvolved Father* and *Control-Focused Father* profiles compared with the *Engaged Supporter Father* profile. Non-Hispanic fathers are more likely to belong to the *Leveled Father* and the *Control-Focused Father* profiles compared with Hispanic fathers. Fathers of Other/Mixed race and White race have different likelihoods of belonging to certain profiles compared with Black/African American fathers. Specifically, Other/Mixed race fathers are more likely to belong to the *Autonomy-Focused Father* profile, while White fathers are more likely to belong to the *Leveled Father*, *Autonomy-Focused Father* and *Uninvolved Father* profiles. Fathers with children aged between 9 and 11 years are more likely to belong to the *Control-Focused Father* profile compared with fathers with children aged between 5 and 8 years. Fathers of girls are more likely to belong to all profiles except the *Engaged Supporter Father* profile compared with fathers of boys. Higher scores on the co-parenting alliance survey are associated with a lower likelihood of belonging to each father profile. Similarly, a higher score on the PEW survey for division of household responsibility is associated with a lower likelihood of belonging to the *Uninvolved Father* profile. The summarised results of the multinomial logistic regression model with significant associations can be found in Table 3, and full results are available in the appendix.

**Table 3.** Significant interactions in the multinomial regression model

Interaction	Estimate	se	OR	95 %CI	*P*-value
Profile (reference: engaged supporter father)					
(Intercept) : leveled father	4·09	1·60	59·61	2·58, 1375·82	0·011
(Intercept) : uninvolved father	4·33	1·72	75·77	2·62, 2194·17	0·012
(Intercept) : control-focused father	7·85	1·69	2564·83	94·19, 69 839·59	0·000
Social determinants of health					
Ethnicity (Reference: Hispanic)					
Non-Hispanic: leveled father	0·99	0·40	2·68	1·22, 5·92	0·015
Non-Hispanic: autonomy-focused father	1·37	0·43	3·92	1·67, 9·19	0·002
Race (Reference: Black/African American)					
Other/Mixed: autonomy-focused father	1·44	0·60	4·22	1·30, 13·66	0·016
White: leveled father	1·02	0·44	2·78	1·17, 6·58	0·020
White: autonomy-focused father	1·10	0·47	3·02	1·20, 7·55	0·018
White: uninvolved father	1·15	0·50	3·15	1·18, 8·38	0·022
Child characteristics					
(Reference: 5–8 years)					
9–11 years: control-focused father	0·85	0·34	2·35	1·20, 4·58	0·012
(Reference Boy)					
Girl: leveled father	0·59	0·30	1·81	1·00, 3·28	0·050
Girl: autonomy-focused father	0·87	0·32	2·40	1·28, 4·48	0·006
Girl: uninvolved father	0·86	0·32	2·37	1·25, 4·47	0·008
Girl: control-focused father	0·92	0·34	2·50	1·28, 4·86	0·007
Family dynamics					
Co-parenting					
Co-parenting : leveled father	−1·05	0·30	0·35	0·19, 0·63	0·001
Co-parenting : autonomy-focused father	−0·71	0·33	0·49	0·26, 0·93	0·029
Co-parenting : uninvolved father	−1·00	0·33	0·37	0·19, 0·69	0·002
Co-parenting : control-focused father	−1·73	0·32	0·18	0·10, 0·33	0·000
Household responsibility					
Household responsibility: uninvolved father	−0·32	0·08	0·72	0·61, 0·85	0·000

se: standard error; CI: confidence intervals.

We observed significant differences between profiles in co-parenting (*P* < 0·001) and household responsibility (*P* = 0·001). When testing for pairwise differences, we found five significant differences in co-parenting: the *Engaged Supporter Father* profile had higher scores compared with *Leveled Father* (*P* < 0·001), *Uninvolved Father* (*P* = 0·002) and *Control-Focused Father* (*P* < 0·001) profiles. Similarly, the *Autonomy-Focused Father* profile had higher scores compared with *Control-Focused Father* (*P* < 0·001) profile, and *Uninvolved Father* profile had higher scores compared with *Control-Focused Father* profile (*P* = 0·003). In the division of household responsibility, we only observed that the *Engaged Supporter Father* profile had higher scores compared with *Uninvolved Father* profile (*P* = 0·001). See Tables 4 and 5 for detailed pairwise comparisons.

**Table 4.** Co-parenting differences between profiles

Profile A				Profile B					
Name	*n*	Mean	sd	Name	*n*	Mean	sd	W	*P*-value
Engaged supporter father	88	4·62	0·56	Leveled father	147	4·33	0·64	0·29	0·000*
Engaged supporter father	88	4·62	0·56	Autonomy-focused father	115	4·44	0·62	0·19	0·079
Engaged supporter father	88	4·62	0·56	Uninvolved father	104	4·41	0·52	0·27	0·002*
Engaged supporter father	88	4·62	0·56	Control-focused father	105	4·06	0·69	0·44	0·000*
Leveled father	147	4·33	0·64	Autonomy-focused father	115	4·44	0·62	0·11	0·71
Leveled father	147	4·33	0·64	Uninvolved father	104	4·41	0·52	0·04	1·000
Leveled father	147	4·33	0·64	Control-focused father	105	4·06	0·69	0·20	0·015
Autonomy-focused father	115	4·44	0·62	Uninvolved father	104	4·41	0·52	0·08	1·000
Autonomy-focused father	115	4·44	0·62	Control-focused father	105	4·06	0·69	0·30	0·000*
Uninvolved father	104	4·41	0·52	Control-focused father	105	4·06	0·69	0·25	0·003*

W: Wilcoxon test; *: Statistically significant at 0·005 significance level after Bonferroni correction. The sample size for this test was *n* 559.

**Table 5.** Household responsibility difference between profiles

Profile A				Profile B					
Name	*n*	Mean (sd)		Name	*n*	Mean (sd)		W	*P*-value
Engaged supporter father	86	4·88	1·97	Leveled Father	140	4·41	1·99	0·10	1·000
Engaged supporter father	86	4·88	1·97	Autonomy-focused father	108	4·54	2·01	0·06	1·000
Engaged supporter father	86	4·88	1·97	Uninvolved father	100	3·63	2·26	0·28	0·001*
Engaged supporter father	86	4·88	1·97	Control-focused father	92	4·50	2·26	0·09	1·000
Leveled father	140	4·41	1·99	Autonomy-focused father	108	4·54	2·01	0·04	1·000
Leveled father	140	4·41	1·99	Uninvolved father	100	3·63	2·26	0·18	0·051
Leveled father	140	4·41	1·99	Control-focused father	92	4·50	2·26	0·02	1·000
Autonomy-focused father	108	4·54	2·01	Uninvolved father	100	3·63	2·26	0·22	0·014
Autonomy-focused father	108	4·54	2·01	Control-focused father	92	4·50	2·26	0·03	1·000
Uninvolved father	100	3·63	2·26	Control-focused father	92	4·50	2·26	0·19	0·077

W: Wilcoxon test; *: Statistically significant at 0·005 significance level after Bonferroni correction. The sample size for this test was *n* 526.

## Discussion

This study had a unique focus on fathers’ parenting practices, and we identified five profiles of fathers based on their combined use of FPP and PAPP: *Engaged Supporter Father* (15·5 %), *Leveled Father* (26·4 %), *Autonomy-Focused Father* (19·3 %), *Uninvolved Father* (18·6 %) and *Control-Focused Father* (20·1 %). We explored the influence of some aspects of social determinants of health, child characteristics and family dynamics in fathers’ profiles with the aim to obtain relevant insights into the factors shaping fatherhood across diverse socio-cultural contexts. We observed significant associations between profile membership and specific categories in race, ethnicity, child characteristics (i.e. age and sex) and family dynamics (i.e. co-parenting and division of household responsibility). However, we did not observe any significant associations between profile membership and any category in education level, annual income and employment status. Moreover, when testing for differences between profiles in co-parenting and household responsibility, we observed significant differences with the *Engaged Supporter Father* reporting higher co-parenting with their partner and greater engagement in household responsibilities than the other profiles. Our findings can inform public health research targeting fathers’ FPP and PAPP, providing a comprehensive analytical framework for two parenting contexts important when promoting positive health trajectories for children. This work contributes to the parenting literature that calls for more person-centered approaches to data analysis rather than variable-centered approaches, which ignore the interdependence between parenting measures^(34)^. This person-centered approach to data analysis examines how variables cluster within individuals, rather than their relationships across a population^(35)^. Our results identify distinct profiles based on response patterns and lay the groundwork for studying individual transitions between profiles using longitudinal data and advanced statistical models (e.g. latent transition analysis).

### Fathers’ profiles based on the combination of FPP and PAPP

Studying FPP and PAPP together allows for a nuanced understanding of parenting practices that directly impact children’s health. Other studies have independently used latent class analysis to identify profiles of parents based on either FPP^(24)^ or PAPP^(8)^ but not in both simultaneously. In the first study examining profiles of PAPP, 618 Canadian parents (51 % mothers) of children aged 5–12 years were surveyed, and four unique latent classes/profiles were identified: *Indifferent*, *Coercive*, *Involved* and *Supportive* ^(8)^. The second study examining profiles of FPP in 799 Canadian parents (50 % mothers) of children aged 5–12 years identified six latent classes/profiles: *Healthy Eating Environment*, *High Engagement*, *Reactive*, *High Structure*, *Controlling* and *Low Engagement* ^(24)^. These studies independently explored the association of the identified classes with physical activity and eating behaviours, concluding that the identification of classes/profiles is theoretically meaningful and crucial for informing family-based interventions. In this study, we identified five profiles based on the differential use across three parenting domains (i.e. autonomy promotion, structure, and control) for both FPP and PAPP. Our profiles shared commonalities in the variability of the domains with the profiles identified in aforementioned studies^(8,24)^. In the three studies, the classes/profiles reflect varying levels of parental engagement, involvement and control. Each study identified a class/profile of parents characterised by (1) low engagement (‘*Indifferent*,’ ‘*Low Engagement*,’ ‘*Uninvolved Father*’), (2) high engagement (‘*Involved*,’ ‘*High Engagement*,’ ‘*Engaged Supporter Father*’) and (3) high controlling parenting (‘*Coercive,*’ ‘*Controlling*,’ ‘*Control-Focused Father*’).

As our approach allowed for a comprehensive perspective, we, interestingly, observed higher variability in fathers’ reported use of PAPP compared with FPP, especially in the autonomy promotion and structure domains. This suggests that fathers may employ a wider array of approaches when encouraging physical activity in their children than when managing their food-related behaviours. Our findings in this sample of fathers align with previous evidence from a study involving 98 parents (59·2 % mothers) that reported results from regression analysis, indicating more consistent results in mothers and for the structure domain of FPP^(36)^. Moreover, the authors discussed gender differences based on the premise that mothers are more involved with feeding children than fathers, suggesting that increasing the involvement of fathers in FPP research and interventions is necessary^(36)^. Regarding PAPP, fathers’ involvement has been recognised from early childhood, particularly through activities like rough-and-tumble play, which foster positive father–child relationships and act as a catalyst for children’s development within the family and the community^(37)^.

In this study, when child characteristics were considered, we observed significant associations between children’s age and sex and profile membership; for instance, fathers with children aged between 9 and 11 years are more likely to belong to the *Control-Focused Father* profile, and fathers of girls are more likely to belong to all profiles except the *Engaged Supporter Father* profile compared with fathers of boys. The significant associations support previous research reporting differences in parenting practices based on child’s sex, suggesting that associations between controlling FPP, extrinsic motivations and fruits/vegetable consumption were observed only in boys^(38)^. Given the observed sex differences and the variability in PAPP that differentiate the profiles, fathers’ lack of engagement especially in providing high involvement, structure and low control may contribute to lower levels of physical activity in girls. Targeting fathers’ engagements may benefit children’s health environments and trajectories, particularly in girls’ physical activity; however, experimental and longitudinal data are needed to support these associations.

### Parenting practices and family dynamics

We observed statistically significant associations and differences in both co-parenting and household responsibility among fathers’ profiles. *Uninvolved Fathers* scored significantly lower in both areas compared with the *Engaged Supporter Fathers*, who had the highest scores. This suggests that *Uninvolved Fathers* may contribute less than their partners, leading to an imbalance in co-parenting and household responsibilities, which may indicate that lack of involvement in FPP and PAPP may be associated with poor family dynamics. Our findings and previous evidence highlight the critical role of family dynamics in shaping parenting practices and suggest that these dynamics should be considered in studies of the FPP and PAPP. For instance, a study modelled parenting styles and co-parenting, conducted among 185 parents (58·4 % mothers) and identified three latent profiles for eating behaviour and weight-related outcomes: *Responsive and Cooperative*, *Minimally Structured* and *Demanding and Competitive* ^(39)^. The authors concluded that considering family dynamics, rather than solely focusing on individual variables in isolation (in their case, parenting styles and co-parenting), was recommended to understand the adaptative nature of parenting and the family^(39)^. The same group conducted two previous studies on feeding co-parenting and concluded that co-parenting influenced both parenting practices and young children’s obesogenic eating behaviours induced by the exacerbation of parents’ psychological distress^(40,41)^. Furthermore, a qualitative study examined the role of thirty-seven fathers in feeding children by examining co-parenting dynamics and outlined conflicting practices (e.g. children’s access to energy-dense, nutrient-poor food) that undermined the practices of each caregiver^(42)^. Considering these inconsistencies in parenting practices, fathers’ involvement and characteristics were recommended for future studies considering children’s behaviour and family dynamics associated with food^(42)^. Interestingly, most of the literature comes from studies focusing on co-parenting and FPP. Further research is needed to explore relationships between family dynamics and parenting practices considering both fathers and mothers; for instance, the relationship between household responsibility and FPP and PAPP should be investigated, as well as how co-parenting influences PAPP.

### Parenting practices and social determinants of health

Fathers, regardless of their background, face unique economic, environmental, racial and cultural challenges. Using social determinants of health to contextualise fathers’ profiles is important, especially given that some people face constraints that influence their behaviours and limit their capacity for good health^(43)^. These constraints contribute to a disproportionate burden of disease and help perpetuate individual and systemic disparities^(14,33)^. The lack of significant associations between fathers’ profiles and socio-economic status variables (e.g. annual income, employment status, and education level) suggests that future studies in parenting practices should consider other social determinants of health, such as acculturation, food insecurity, healthcare access and quality, neighbourhood and environment and community and cultural context. Neighbourhood characteristics, for instance, have been linked to parental disciplinary practices, with parents in more dangerous neighbourhoods reporting stricter parenting to ensure their children’s safety^(44)^. Thus, although we measured a few social determinants of health, future studies will need to assess the complexity of paternal roles beyond traditional indicators of socio-economic status^(14,45)^.

Disentangling why race and ethnicity impact parenting requires examining the macro-level forces that reinforce inequities and lead to variations in parenting practices. Although our study identified some differences in parenting based on race and ethnicity, exploring these differences qualitatively is crucial for developing culturally sensitive interventions. Without this deeper understanding, interventions may lack the contextual foundation needed for effectiveness. With this in mind, we aimed to contextualise our findings by acknowledging the heterogeneity within our sample and recognising that we may miss relevant culturally and contextually grounded information. Having a diverse sample of fathers allowed us to identify that self-identifying as non-Hispanic, White or Other/Mixed race, independently, was associated with profile membership for the *Leveled Father*, *Autonomy-Focused Father* and *Uninvolved Father* profiles compared with fathers who self-identified as Hispanic or Black/African American. This can not only be explained by the variability in the use of parenting practices but also aligns with the body of literature suggesting that race and ethnicity can shape parenting^(14,24)^. However, differences in parenting can be attributed to various socio-cultural factors, rather than relying solely on categories of race and ethnicity, which may not fully encompass the diversity within communities and can contribute to the perpetuation of disparities^(33)^. Indeed, evidence suggests that economic disparities and cultural differences in feeding norms and practices across racial groups could affect the use of FPP^(46,47)^. For instance, a study conducted with 3709 parents (62 % mothers) explored how parents of adolescents attempted to regulate their children’s eating behaviours and concluded that controlling FPP (i.e. pressure to eat and intake restriction) is common among parents in racial and ethnic minority subgroups^(48)^. Similarly, cultural norms shaped by race and ethnicity and social context influence PAPP resulting in a variation of children’s physical activity behaviours^(49)^. In this study, we did not find significant associations between either race or ethnicity and the control-focused father profile. This finding may help prevent stereotypes that associate racial and ethnic minority subgroups with controlling or coercive parenting practices.

### Limitations and strengths and future research

This study has some limitations. We do not provide evidence of causality due to the cross-sectional nature of the data and the lack of outcomes in the children. Self-selection bias is also an issue, as individuals who chose to complete the online survey may differ from those who did not. Moreover, most of our sample resided in the city of Houston (66·1 %) and the state of Texas (91·9 %). Our results may not be generalisable to fathering children in other age ranges different from our sample (age 5–11 years). Additional social determinants of health could have been included in the study; for instance, we did not consider food insecurity, acculturation, neighbourhood, community context and macro-level factors such as structural practices that perpetuate inequities, which can shape specific parenting practices. Regarding demographic variables, fathers’ height and weight were self-reported and we asked for children’s age categories instead of date of birth which may introduce errors. Finally, we may have lost statistical power by including variables with multiple categories in the model.

We also acknowledge the strengths of our study. First, we used data collected using empirically tested instruments. We analysed a relatively large and diverse sample of fathers, which allowed us to bring social determinants of health into the statistical model and the discussion. For example, we used multiple race categories to account for more variability compared with a dichotomous white/non-white variable and followed recommended reporting practices for race and ethnicity^(33)^. We also completed a comprehensive analysis using a person-centered approach. Accounting for family dynamics allowed us to get insights into how co-parenting and household responsibility may explain group membership. Future research in fathers’ FPP and PAPP should continue using a person-centered approach in experimental or longitudinal studies.

### Conclusion

The combination of FPP and PAPP allowed us to identify five profiles of fathers: *Engaged Supporter Father, Leveled Father, Autonomy Focused Father, Uninvolved Father* and *Control-Focused Father*. Considering the interplay of fathers’ FPP and PAPP may enhance assessments for a holistic understanding of children’s health environments to advance public health. This understanding underscores the need for targeted, person-centered, research that addresses the complex interplay of factors affecting parenting. Recognising the characteristics and differences between fathers’ profiles allows for tailored interventions to address family dynamics and needs. Such interventions may contribute to the promotion of children’s health and well-being across diverse populations.

Race, ethnicity, child characteristics and family dynamics may help explain fathers’ profiles. The study of the intersection of father profiles with social determinants of health must support the notion that ‘one size does not fit all’ to avoid perpetuating stereotypes. Providing targeted support, contextualised within cultural nuances, can help address profiles that may contribute to health disparities in their children. Moreover, the fathers’ profiles can influence family dynamics, affecting the social support network at the family level, and can strengthen family bonds that may serve as a protective factor that buffers against chronic stress induced by social determinants of health and health disparities^(50)^. As children learn from parenting practices, early established healthy habits are more likely to persist into adulthood. Fathers’ profiles based on FPP and PAPP can have a long-term impact on children’s health trajectories.

## Financial support

This research was funded by the National Heart, Lung and Blood Institute of the National Institutes of Health (grant number R34HL131726). This work also is a publication of the United States Department of Agriculture (USDA/ARS) Children’s Nutrition Research Center, Department of Pediatrics, Baylor College of Medicine, Houston, TX and has been funded in part with federal funds from the USDA/ARS (cooperative agreement number 58–3092–5–001). The contents of this work are solely the responsibility of the authors and do not necessarily represent the official views of the USDA. Louise C. Mâsse received salary support from the BC Children’s Hospital Research Institute.

## Conflict of interest

There are no conflicts of interest.

## Authorship

J.A.J-G.: Statistical planning and analysis; writing – original draft preparation and editing. L.C.M.: Statistical planning; writing – reviewing and editing. R.L.N.: Writing – reviewing and editing. S.M.M.: Statistical planning; writing – reviewing and editing. A.B.: Data collection; writing – reviewing and editing. T.M.O’C.: Idea inception; writing – original draft preparation, reviewing and editing.

## Ethics of human subject participation

This study was conducted according to the guidelines laid down in the Declaration of Helsinki, and all procedures involving research study participants were approved by the Institutional Review Board (IRB) at Baylor College of Medicine (protocol: H-38237). Written informed consent was obtained from all subjects/patients.

Data availability statement: The de-identified data can be made available under reasonable requests for the IRB-approved intended uses and with an appropriate formal institutional data-sharing agreement established.

## Supplementary material

For supplementary material accompanying this paper visit https://doi.org/10.1017/S1368980025000278

## Supporting information

Jimenez-Garcia et al. supplementary material Jimenez-Garcia et al. supplementary material
