# Supplementary material for: Exploring profiles of fathers integrating food and physical activity parenting practices
Source: Public Health Nutr. 2025 Mar 17;28(1):e58. doi: 10.1017/S1368980025000278 (PMC12086733; doi:10.1017/S1368980025000278)
Supplement: Jimenez-Garcia et al. supplementary material [file S1368980025000278sup001.docx]

**Appendix 1.** Complete results of the multinomial logistic regression model

| **Interaction** | **Estimate (SE)** | **Odds Ratio [95%CI]** | **p-value** |  |
| --- | --- | --- | --- | --- |
| **Profile (Reference: Engaged Supporter Father)** |  |  |  |  |
| (Intercept) : Leveled Father | 4.09 (1.60) | 59.61 [2.58 - 1375.82] | 0.011 | * |
| (Intercept) : Autonomy-Focused Father | 1.93 (1.73) | 6.88 [0.23 - 203.14] | 0.264 |  |
| (Intercept) : Uninvolved Father | 4.33 (1.72) | 75.77 [2.62 - 2194.17] | 0.012 | * |
| (Intercept) : Control-Focused Father | 7.85 (1.69) | 2564.83 [94.19 - 69839.59] | 0.000 | * |
| **Social Determinants of Health** |  |  |  |  |
| **Income (Reference: More than 47K)** |  |  |  |  |
| Less than 25K : Leveled Father | 0.31 (0.54) | 0.73 [0.25 - 2.11] | 0.561 |  |
| Less than 25K : Autonomy-Focused Father | 0.05 (0.60) | 0.95 [0.29 - 3.08] | 0.932 |  |
| Less than 25K : Uninvolved Father | 0.29 (0.57) | 0.75 [0.24 - 2.27] | 0.605 |  |
| Less than 25K : Control-Focused Father | 0.26 (0.61) | 0.77 [0.24 - 2.54] | 0.670 |  |
| 25K-47K : Leveled Father | 0.25 (0.42) | 1.29 [0.57 - 2.91] | 0.540 |  |
| 25K-47K : Autonomy-Focused Father | 0.10 (0.45) | 1.11 [0.46 - 2.68] | 0.822 |  |
| 25K-47K : Uninvolved Father | -0.11 (0.45) | 0.89 [0.37 - 2.17] | 0.803 |  |
| 25K-47K : Control-Focused Father | 0.54 (0.45) | 1.71 [0.70 - 4.16] | 0.239 |  |
| **Education Level (Reference: University Degree)** |  |  |  |  |
| Non-University Degree : Leveled Father | -0.07 (0.36) | 0.93 [0.46 - 1.89] | 0.850 |  |
| Non-University Degree : Autonomy-Focused Father | -0.25 (0.38) | 0.78 [0.37 - 1.64] | 0.504 |  |
| Non-University Degree : Uninvolved Father | 0.05 (0.39) | 1.05 [0.49 - 2.26] | 0.900 |  |
| Non-University Degree : Control-Focused Father | -0.38 (0.41) | 0.68 [0.31 - 1.53] | 0.357 |  |
| **Employment Status (Reference Employed 40hrs)** |  |  |  |  |
| Not Employed : Leveled Father | -0.07 (0.58) | 0.93 [0.30 - 2.92] | 0.899 |  |
| Not Employed : Autonomy-Focused Father | 0.38 (0.57) | 1.46 [0.48 - 4.51] | 0.506 |  |
| Not Employed : Uninvolved Father | 0.82 (0.59) | 2.26 [0.71 - 7.20] | 0.168 |  |
| Not Employed : Control-Focused Father | 0.21 (0.62) | 1.23 [0.37 - 4.14] | 0.736 |  |
| Less than 40hrs : Leveled Father | -0.53 (0.52) | 0.59 [0.21 - 1.61] | 0.300 |  |
| Less than 40hrs : Autonomy-Focused Father | -1.19 (0.62) | 0.31 [0.09 - 1.03] | 0.055 |  |
| Less than 40hrs : Uninvolved Father | -0.46 (0.59) | 0.63 [0.20 - 2.01] | 0.437 |  |
| Less than 40hrs : Control-Focused Father | -1.26 (0.65) | 0.28 [0.08 - 1.01] | 0.052 |  |
| More than 40hrs : Leveled Father | 0.36 (0.38) | 1.43 [0.67 - 3.03] | 0.351 |  |
| More than 40hrs : Autonomy-Focused Father | -0.35 (0.43) | 0.71 [0.30 - 1.64] | 0.417 |  |
| More than 40hrs : Uninvolved Father | 0.22 (0.41) | 1.25 [0.56 - 2.81] | 0.587 |  |
| More than 40hrs : Control-Focused Father | -0.44 (0.48) | 0.64 [0.25 - 1.64] | 0.354 |  |
| **Ethnicity (Reference: Hispanic)** |  |  |  |  |
| Non-Hispanic : Leveled Father | 0.99 (0.40) | 2.68 [1.22 - 5.92] | 0.015 | * |
| Non-Hispanic : Autonomy-Focused Father | 1.37 (0.43) | 3.92 [1.67 - 9.19] | 0.002 | * |
| Non-Hispanic : Uninvolved Father | 0.73 (0.43) | 2.08 [0.89 - 4.85] | 0.090 |  |
| Non-Hispanic : Control-Focused Father | -0.07 (0.46) | 0.93 [0.38 - 2.32] | 0.882 |  |
| **Race (Reference: African American)** |  |  |  |  |
| Asian : Leveled Father | 0.44 (0.73) | 1.55 [0.37 - 6.50] | 0.548 |  |
| Asian : Autonomy-Focused Father | 0.23 (0.78) | 1.26 [0.27 - 5.82] | 0.768 |  |
| Asian : Uninvolved Father | 0.33 (0.83) | 1.40 [0.28 - 7.04] | 0.685 |  |
| Asian : Control-Focused Father | 0.24 (0.76) | 1.27 [0.29 - 5.59] | 0.754 |  |
| Other/Mixed : Leveled Father | 0.87 (0.57) | 2.38 [0.78 - 7.24] | 0.126 |  |
| Other/Mixed : Autonomy-Focused Father | 1.44 (0.60) | 4.22 [1.30 - 13.66] | 0.016 | * |
| Other/Mixed : Uninvolved Father | 1.11 (0.62) | 3.05 [0.90 - 10.28] | 0.072 |  |
| Other/Mixed : Control-Focused Father | 0.03 (0.62) | 1.03 [0.30 - 3.50] | 0.962 |  |
| White : Leveled Father | 1.02 (0.44) | 2.78 [1.17 - 6.58] | 0.020 | * |
| White : Autonomy-Focused Father | 1.10 (0.47) | 3.02 [1.20 - 7.55] | 0.018 | * |
| White : Uninvolved Father | 1.15 (0.50) | 3.15 [1.18 - 8.38] | 0.022 | * |
| White : Control-Focused Father | -0.14 (0.49) | 0.87 [0.33 - 2.28] | 0.779 |  |
| **Child Characteristics** |  |  |  |  |
| **(Reference: 5-8 years)** |  |  |  |  |
| 9-11 years : Leveled Father | 0.15 (0.31) | 1.16 [0.63 - 2.13] | 0.635 |  |
| 9-11 years : Autonomy-Focused Father | 0.16 (0.33) | 1.17 [0.61 - 2.24] | 0.629 |  |
| 9-11 years : Uninvolved Father | -0.05 (0.34) | 0.96 [0.49 - 1.88] | 0.895 |  |
| 9-11 years : Control-Focused Father | 0.85 (0.34) | 2.35 [1.20 - 4.58] | 0.012 | * |
| **(Reference Boy)** |  |  |  |  |
| Girl : Leveled Father | 0.59 (0.30) | 1.81 [1.00 - 3.28] | 0.050 | * |
| Girl : Autonomy-Focused Father | 0.87 (0.32) | 2.40 [1.28 - 4.48] | 0.006 | * |
| Girl : Uninvolved Father | 0.86 (0.32) | 2.37 [1.25 - 4.47] | 0.008 | * |
| Girl : Control-Focused Father | 0.92 (0.34) | 2.50 [1.28 - 4.86] | 0.007 | * |
| **Family Dynamics** |  |  |  |  |
| **Co-Parenting** |  |  |  |  |
| Co-Parenting : Leveled Father | -1.05 (0.30) | 0.35 [0.19 - 0.63] | 0.001 | * |
| Co-Parenting : Autonomy-Focused Father | -0.71 (0.33) | 0.49 [0.26 - 0.93] | 0.029 | * |
| Co-Parenting : Uninvolved Father | -1.00 (0.33) | 0.37 [0.19 - 0.69] | 0.002 | * |
| Co-Parenting : Control-Focused Father | -1.73 (0.32) | 0.18 [0.10 - 0.33] | 0.000 | * |
| **Household Responsibility** |  |  |  |  |
| Household Responsibility : Leveled Father | -0.12 (0.08) | 0.89 [0.77 - 1.04] | 0.131 |  |
| Household Responsibility : Autonomy-Focused Father | -0.09 (0.08) | 0.91 [0.78 - 1.07] | 0.275 |  |
| Household Responsibility : Uninvolved Father | -0.32 (0.08) | 0.72 [0.61 - 0.85] | 0.000 | * |
| Household Responsibility : Control-Focused Father | -0.13 (0.09) | 0.88 [0.74 - 1.04] | 0.123 |  |
| * : significant at 0.05 level; SE : Standard Error |  |  |  |  |
